# Interventions for Pediatric Sepsis and Their Impact on Outcomes: A Brief Review

**DOI:** 10.3390/healthcare7010002

**Published:** 2018-12-28

**Authors:** Laura A. Watkins

**Affiliations:** Department of Pediatrics/Critical Care, School of Medicine and Dentistry, University of Rochester Medical Center, 601 Elmwood Ave, Box 667, Rochester, NY 14642, USA; Laura_Watkins@urmc.rochester.edu

**Keywords:** sepsis, pediatrics, outcomes

## Abstract

In the current era, pediatric sepsis remains a public health problem of significant prevalence and impact. With mortality rates practically unchanged over the years, this review hopes to briefly summarize the epidemiology and the current interventions for pediatric sepsis and point towards possible areas of improvement. Most pediatric studies of sepsis are either small, retrospective or observational. Given information technology spreading across country, and a stronger presence of clinical networks, development of multicenter prospective studies over the next decade should enable better treatments for pediatric sepsis, and improved outcomes.

## 1. Introduction

### 1.1. Sepsis Severity and Prevalence

Sepsis is a major cause of death and morbidity in the United States and throughout the world. It is estimated to occur among children in the US at a rate of 158.7 cases per 100,000 children [1]. Of these, over one third requires hospitalization with total healthcare costs exceeding 14 billion dollars annually [2]. Sepsis is the tenth leading cause of death among children in the US, with an overall mortality of 4%–10%, reaching as high as 24% for children admitted to the Pediatric Intensive Care Unit (PICU) [3]. For children with access to medical care, outcomes are similar in resource-rich continents (North America, Europe, Australia/New Zealand), compared to resource-poor parts of the world (Asia, Africa and South America) where PICU mortality is 23% versus 29% and hospital mortality is 24% versus 31% [3]. Worldwide, approximately half of pediatric deaths are due to infection [4,5] and 29%–40% of children admitted to the PICU with sepsis will ultimately die. In the last few years, an increase in the prevalence of sepsis has been observed. This change likely reflects an increase in sepsis surveillance, an expanding population of vulnerable children with chronic morbidities, and increasing rates of multidrug resistant organisms and opportunistic infection [3,4].

### 1.2. Definition of Sepsis

Sepsis is a life-threatening organ dysfunction caused by a dysregulated host response to infection [6]. Adult clinical criteria for establishing a diagnosis in the sepsis continuum has recently been modified [6] and this change has been criticized [7]. To further complicate matters, pediatric sepsis criteria still employ older definitions based upon Systemic Inflammatory Response Syndrome (SIRS) definitions [8]. Whereas the adult (Sepsis-3) definitions have narrowed the sepsis continuum to sepsis and septic shock, the older pediatric definitions still employ the categories of sepsis, severe sepsis and septic shock, defining each, respectively, as SIRS in response to infection, sepsis with organ dysfunction and/or tissue hypoperfusion and sepsis with hypotension despite adequate resuscitation. A revision of pediatric criteria is expected in the near future.

## 2. Sepsis in Chronically Ill versus Previously Healthy Children

Refractory shock is the most common cause of death in severe pediatric sepsis (34%), followed by multiple organ dysfunction syndrome (MODS) after shock recovery (27%), neurologic injury (19%) and respiratory failure (9%) [9]. Recent studies of death in children with sepsis reveal two distinct populations. The first and most common group are chronically ill children [4], typically admitted to PICU from the pediatric ward. The comorbidities in these patients, such as organ/stem cell transplant, malignancy, renal disease and other hematologic-oncologic conditions are associated with an increase of the odds ratio (OR) of mortality of 1.5–2.0, compared to the baseline population [3]. In these children, death usually occurs after at least one week of PICU care, after unrelenting MODS that finally leads to a decision to withdraw artificial support or to withhold life-sustaining therapies [3,9]. The second, much smaller population of patients who die from sepsis experience a more rapid decline: they represent one third of the deaths and they die during the first three days of the PICU course. These are previously healthy children who are usually admitted to PICU through the emergency department [9]. It is unclear if differences in the course of sepsis between these 2 populations are attributable to different pathophysiology, or reduced resilience in the children with chronic diseases. More research is needed.

## 3. Chronic Sepsis Outcomes

The examination of pediatric sepsis outcomes must include an analysis not only of mortality, but also of disability, change in quality of life and the development of new or progressive organ dysfunction. Forty percent of pediatric patients with severe sepsis will develop dysfunction of at least one additional organ and 58% will develop MODS, usually in the first week of illness [10]. Among pediatric sepsis survivors, disability occurs in approximately 34–48%, with ~17% of children having moderate or severe disability [3,11]. Overall, 38% of pediatric patients diagnosed with severe sepsis or septic shock either die or are discharged in a moderately to severely disabled state [3].

## 4. Current Treatment Strategies and Gaps in Knowledge

Pediatric sepsis outcomes depend on multiple factors, including the complex interaction between the infectious agent and host defense mechanisms, prompt recognition and adequate medical care. Consensus guidelines support instituting prompt, targeted therapies, early anti-microbial treatment and support of organ dysfunction [12,13]. Clinical attention must focus on the entire spectrum of management, from recognition to initial resuscitation to stabilization, organ failure resolution and functional outcomes.

Several aspects of clinical practice need further investigation to optimize pediatric sepsis outcomes (Table 1).

### 4.1. Early Sepsis Recognition

SIRS criteria in pediatrics are sensitive (95%), but not specific (15%). Thus, many children meeting the definition for sepsis do not actually have the condition. Although a recognition bundle has been advocated [13], and is even required by law in some states including New York (10 CRR-NY 405.4), it is challenging to accurately identify pediatrics patients with sepsis without a true gold standard (i.e., with a high positive and negative predictive value).

### 4.2. Early Goal Directed Therapy (EGDT)

Following the publishing of River’s study in 2001 [14], Early Goal Directed Therapy (EGDT) became standard practice for the treatment of adult sepsis for many years. Further studies using EGDT were unable to show improved outcomes, although two relatively small pediatric studies have shown improved mortality by targeting resuscitation to the goal of superior vena cava oximetry (SvcO2) above 70 [15,16].

### 4.3. Protocolized Medicine

Sepsis protocols or “bundles” have been associated with improved sepsis outcomes for children [17]. In one study, the odds of death were five times higher in children who did not receive bundle-compliant care. However, this study was performed over seven years and historic improvement in attention to details of care likely confounded the findings [18]. Another study found that hospital length of stay was reduced for pediatric septic patients who received treatment following the Pediatric Advanced Life Support (PALS) recommendations [19].

### 4.4. Bedside Ultrasonography and Echocardiography

Interest in the use of ultrasound to guide sepsis therapy is growing. Recent studies suggest that bedside echocardiography in pediatric septic shock can be used to improve mortality and reduce shock reversal time [20,21]. One caveat is that the categorization of fluid responsiveness in both of these studies has no support in the current literature, and arguably. patients received smaller quantities of fluid than they would otherwise. By using bedside echocardiography, the study patients in one of these trials were four-fold less likely to show signs of fluid overload by 24 h, and their central venous pressure (CVP) was significantly lower [21]. Sepsis-induced myocardial dysfunction is increasingly being recognized in pediatric sepsis [22,23,24], however, a standard evaluation accepted by the pediatric bedside ultrasound community has yet to be published. Strain echo may be an effective tool for early diagnosis of sepsis-induced myocardial systolic dysfunction in pediatric septic shock [25], however, translation of this method to routine bedside management of pediatric sepsis is not feasible, because of equipment cost and training requirements. Of the simple bedside measurements, mitral annular plane systolic excursion (MAPSE) was found to be an independent predictor of mortality in children with septic shock, in a small study [26].

### 4.5. Fluid Resuscitation

#### 4.5.1. Fluid Administration

Giving fluids is clearly indicated in the treatment of pediatric sepsis [12,13]. Although both Surviving Sepsis and Pediatrics Advanced Life Support guidelines have recommendations regarding fluid resuscitation, the precise volume for a given patient that would help and not harm is challenging to determine. Fluid is certainly beneficial in hypovolemic shock; upon presentation to care, septic children have a component of dehydration that would benefit from fluid administration. However, in the situation of capillary leak, the administered fluid may not remain inside the vessels for long [27,28]. An emerging body of evidence suggests that aggressive fluid resuscitation leads to severe tissue edema, with compromise of organ function and increased morbidity and mortality [29,30,31]. This problem is exacerbated by malnutrition and severe anemia, which are common in many parts of the developing world, as shown by the Fluid Expansion as Supportive Therapy (FEAST) study [32]. Goal directed therapy is essential, and treatments to prevent or reverse excess fluid accumulation should be employed. A conceptual change is needed, in which fluid responsiveness should not be assumed to represent a fluid requirement. Striving to find the right fluid balance that is beneficial and not harmful for our sickest septic children must take center stage [33,34,35].

#### 4.5.2. Type of Fluid Used for Resuscitation

The appropriate resuscitation fluid remains an area of controversy and ongoing research. American College of Critical Care Medicine (ACCM) Clinical Practice Parameters for hemodynamic support of pediatric and neonatal shock refers to a crystalloid, a colloid or a blood product as a choice of fluid, depending on the clinical situation (although mostly crystalloids (normal saline or Ringer’s lactate solution), with albumin in conditions of decreased plasma oncotic pressure, blood if needed to obtain a ScvO2 above 70 in patients with hemoglobin under 10, and fresh frozen plasma (FFP) infusion in case of coagulopathy). Normal saline, although isotonic, is far from “normal” in physiologic composition, given its high sodium and chloride concentration relative to plasma, as well as its strong ion difference of 0. In healthy adult volunteers, administration of excessive normal saline was associated with decreased renal perfusion [36]. Hyperchloremia during the first week of septic shock in children is associated with an increased risk of mortality [37]. In a retrospective study, balanced fluids, such as Ringer’s lactate and plasma-lyte, were compared to sodium chloride, when used exclusively during the first 72 h of resuscitation. Balanced fluids were associated with improved survival in the 1–4 years old severe sepsis group (OR 0.76) with decreased overall development of acute kidney injury (OR 0.82) [38].

#### 4.5.3. Rapidity of Fluid Resuscitation

The rapidity of fluid resuscitation has not been deeply studied. A recent randomized controlled trial concluded that faster bolus administration (e.g., >5–10 min versus >15–20 min) increased the risk of intubation and mechanical ventilation [39]. However, the results of this paper have been intensely debated [40,41], and the question of how fast we should administer the fluid boluses in pediatric septic patients remains unclear.

### 4.6. Medications

#### 4.6.1. Type and Timing of Antibiotics

Fast initiation of antibiotic therapy is a mainstay of sepsis treatment. A recent pediatric study reaffirms the Surviving Sepsis campaign goals, showing that a 3 h delay is associated with increased mortality and organ dysfunction [42]. Similarly, a retrospective observational single center study reported that administration of antibiotics in <1 h or >3 h from sepsis recognition was associated with increased 1 year mortality in severe cases of pediatric sepsis [43]. Children with complex medical conditions have a greater risk for sepsis and these patients may be infected with multi-drug resistant organisms; hence, the use of appropriate broad-spectrum antibiotics is important in this population [44]. The optimal duration of antibiotic treatment in pediatric sepsis is also uncertain [45]. Collaborative and evidence-based strategies for antibiotic de-escalation in the pediatric critical care environment are needed [45,46].

#### 4.6.2. Choice of Vasopressor Therapy

Compared to adults, children with septic shock have great variability in the relationship between cardiac output and systemic vascular resistance. Therefore, the choice of vasoactive support for children varies much more than for adults [47]. Recent studies have suggested that epinephrine is superior to dopamine as the primary vasoactive drug to treat pediatric septic shock [48,49]. Vasodilatory shock is a pattern more often found in the adult or older child and, extrapolating from adult studies, norepinephrine is commonly the primary vasoactive medication in this situation [12,34]. An international study examining the choice of vasoactive medications for children with severe sepsis and septic shock showed that epinephrine, norepinephrine, dopamine and milrinone were used in 43%, 42%, 32% and 30%, respectively [3]. Other vasoactive medications were utilized in only 3% of pediatric patients. As in adult sepsis therapy, there was a trend toward greater use of norepinephrine in older children [3].

#### 4.6.3. Corticosteroids

Corticosteroids are used in approximately 45% of children with severe sepsis and septic shock [3]. Furthermore, an overwhelming majority of pediatric intensivists believe that corticosteroids have a significant role in the treatment of pediatric sepsis [50]. However, despite the frequency of steroid use proof of the value of steroids for pediatric sepsis remains elusive, and no major randomized controlled trials have been published. A systematic review and meta-analysis demonstrated the difficulty of investigating the use of steroids in pediatric sepsis and the meta-analysis failed to confirm the effectiveness of this class of medications [51]. Intensive controversy surrounds the risks and benefits of corticosteroids. An observational study noted that the administration of steroids is independently associated with increased odds of death (adjusted OR of 1.5) [3]. A retrospective cohort study of pediatric patients with catecholamine dependent septic shock who received stress doses of steroids had greater PICU and hospital length of stay and fewer ventilator free days if the random cortisol level was <18 μg/dl; they had higher PICU mortality and ventilator and vasopressor free days if the random cortisol level was >18 μg/dl [52]. A large, multicenter, randomized, controlled clinical trial would help to resolve this question, if such a study could gain acceptance [53]. As is the case with any serious acute medical condition, patients with adrenal insufficiency should receive stress-dose steroids when they are sick and suspected of having sepsis [12].

### 4.7. Other Interventions

#### 4.7.1. Source Control

Despite greater severity and worse compliance with resuscitation bundles, mortality was lower in adult septic patients who underwent source control compared to those who did not [54]. There is a paucity of literature on this subject in pediatric sepsis.

#### 4.7.2. Adjuvant Therapies

Other proposed therapies, including insulin, g/gm-csf, immunoglobulins, plasma exchange and extra-corporeal support, are uncommonly used in the treatment of pediatric sepsis [55,56,57]. Additional novel combination therapies such as hydrocortisone, thiamine and vitamin C [58] have not yet been studied in children.

### 4.8. Post-ICU Critical Care Syndrome-Pediatrics (PICS-p)

PICS-p is a condition that entails the long-term effects of sepsis on PICU survivors [59]. Research on the long-term mortality of pediatric patients with sepsis is scarce. More children are discharged with disability than die of sepsis, thus, it is important to prevent and treat the chronic effects of critical care illness. ICU interventions must not only focus on acute illness, but must also consider the physical, cognitive, emotional and social health of the critically ill child both in and beyond the PICU [60]. Important considerations include choice of sedative medication, monitoring and treatment of delirium, early mobilization, appropriate liberation from mechanical ventilation and avoidance of restraints and coercion.

## 5. Conclusions

In the current era, pediatric sepsis remains a life-threatening disease whose outcomes can be devastating. Because of the many gaps in the pediatric-specific literature, therapeutic interventions are often based on adult studies and expert opinion, rather than concrete evidence. Children with sepsis would benefit from high-quality research on improved interventions. Common challenges for pediatric research in general are the small number of patients with a specific disease at a single institution, limited availability of pediatric grant funding and the complexity of human subjects review and consent for research on children [61,62]. Facilitation of research by clinical networks such as Pediatric Acute Lung Injury and Sepsis Investigators (PALISI), coupled with better access to patient data using protocols for extracting research data from electronic health records, should make the future of children with sepsis brighter in the next decade.

## Figures and Tables

**Table 1:** Current treatment strategies and areas needing more research.

Intervention	Research Gaps
Early sepsis recognition	Define sepsis more accurately
Early goal directed therapy	Larger studies needed, new goals need to be identified
Protocolized medicine	Evidence supportive; likely difficult to study given mandatory protocolized medicine for sepsis
Bedside ultrasonography and echocardiography	Larger studies are needed using appropriate protocols
Fluid resuscitation	More research needed regarding the right amount, right type and rapidity of the bolus administration
Type and timing of antibiotics	Tailoring for patients with chronic disease and sepsis; De-escalation and appropriate length of antibiotic therapy needs further research
Choice of vasopressor therapy	Tailoring to different clinical presentations and use of point-of-care imaging
Corticosteroids	Conflicting evidence; no large randomized controlled trials published
Source control	No studies in pediatrics
Adjuvant therapies	Clinical research on this approach to therapy is very difficult because use is uncommon and patients are very complex
Post-ICU critical care syndrome-pediatrics	Very little research has been done
